# Development of Ionic Liquid Modified Disposable Graphite Electrodes for Label-Free Electrochemical Detection of DNA Hybridization Related to *Microcystis* spp.

**DOI:** 10.3390/s150922737

**Published:** 2015-09-09

**Authors:** Ceren Sengiz, Gulsah Congur, Arzum Erdem

**Affiliations:** Faculty of Pharmacy, Analytical Chemistry department, Ege University, Bornova, 35100 Izmir, Turkey; E-Mails: ceren.sengiz@gmail.com (C.S.); gulsah.congur@gmail.com (G.C.)

**Keywords:** ionic liquid, nucleic acid hybridization, *Microcystis* spp., electrochemical DNA biosensor, pencil graphite electrode

## Abstract

In this present study, ionic liquid (1-butyl-3-methylimidazolium hexafluorophosphate (IL)) modified pencil graphite electrode (IL-PGEs) was developed for electrochemical monitoring of DNA hybridization related to *Microcystis* spp. (MYC). The characterization of IL-PGEs was performed using microscopic and electrochemical techniques. DNA hybridization related to MYC was then explored at the surface of IL-PGEs using differential pulse voltammetry (DPV) technique. After the experimental parameters were optimized, the sequence-selective DNA hybridization related to MYC was performed in the case of hybridization between MYC probe and its complementary DNA target, noncomplementary (NC) or mismatched DNA sequence (MM), or and in the presence of mixture of DNA target: NC (1:1) and DNA target: MM (1:1).

## 1. Introduction

Nucleic acid analysis using biosensing strategies has been an attracting topic in many fields including gene analysis, clinical disease diagnosis, biological, environmental, pharmaceutical and forensic applications [1,2,3,4,5,6,7,8]. Several detection techniques such as optic [9,10], acoustic [11], quartz crystal microbalance [12], and different electrochemical techniques [1,2,4,5,6,13,14,15,16,17,18,19,20,21,22,23,24,25,26,27,28,29,30,31,32,33] have been used for nucleic acid analysis. 

Effective immobilization of oligonucleotides onto the surface of analysis platform has been one of the key features for the successful design of biosensor platforms [1,2,4,5,6,13,14,15,16,17,18,19,20,21,22,23,24,25,26,27,28,29,30,31,32,33]. Ionic liquids (ILs) are known as low-melting organic salts and mostly liquid at room temperature. Due to their unique properties such as low measurable vapor pressure, high thermal stability and conductivity, having good solvating properties, non-volatility, low toxicity and biocompatibility, they have been used in different fields including development of electrochemical biosensors [13,21,22,23,24,25,26,27,31,32,33]. Ren *et al.* [32] developed a chronocoulometric DNA sensor that based on polyaniline nanotubes (PANINTs) and ionic liquid (IL) doped screen-printed electrode. Eksin *et al.* [27] developed the chitosan/ionic liquid modified pencil graphite electrodes (CHIT-IL-PGEs) for the first time in order to perform enhanced electrochemical monitoring of nucleic acid, and the interaction of the anticancer drug, Mitomycin C (MC) and calf thymus double stranded DNA (dsDNA). She *et al.* [31] investigated the detection of hydroquinone on the carbon paste electrode enhanced by the hydrophobic IL.

The microcystins (MYC), which are a family of cyclic polypeptides produced by different species of cyanobacteria, are among the most frequently detected in fresh waters, and produce potent toxic effects. Consumption of contaminated waters and food (agricultural products, fish, prawns, and mollusks), can cause human diseases such as gastroenteritis and related diseases, allergic and irritation reactions, liver cancer and colorectal cancers in consequence of exposure to low MYC concentrations through dermal exposure and inhalation [14,34,35,36,37,38,39,40,41].

To minimize the risk to human health through exposure to microcystins, there is a requirement for sensitive and reliable methods capable of detecting this class of toxins in a wide range of sample matrices [14,34,35,36,37,38,39,40,41]. Yan *et al.* [39] developed an electrochemical DNA biosensor for the rapid detection of specific gene related to MYC based on immobilizing a DNA probe, complementary to a specific gene sequence related to MYC on a gold electrode through specific adsorption by using methylene blue and ruthenium bipyridine as electrochemical hybridization indicators. Erdem *et al.* [14] investigated the electrochemical label-free and indicator based monitoring of DNA hybridization related to MYC DNA sequence via multiwalled carbon nanotube (MWCNT) modified screen printed graphite electrodes (SPEs). 

In our study, a simple, practical and low-cost electrochemical DNA biosensor was developed by using ionic liquid (1-butyl-3-methylimidazolium hexafluorophosphate (IL)) disposable pencil graphite electrodes (PGEs). The microscopic and electrochemical characterization of IL modified PGEs was performed. Then, IL-PGEs were utilized for electrochemical monitoring of sequence selective DNA hybridization related to *Microcystis* spp. (MYC) without using any external hybridization indicator. Under the optimum experimental conditions, the selectivity of the electrochemical biosensor was tested in the presence of noncomplementary (NC) or mismatched DNA sequence (MM), or and in the presence of mixture of DNA target: NC (1:1) and DNA target: MM (1:1). 

## 2. Experimental Section

### 2.1. Apparatus

The electrochemical measurements including differential pulse voltammetry (DPV), cyclic voltammetry (CV) and electrochemical impedance spectroscopy (EIS) were performed by using AUTOLAB-PGSTAT 302 electrochemical analysis system supplied with GPES 4.9007 software package (Eco Chemie, Utrecht, The Netherlands). 

A conventional three-electrode system was consisted of disposable pencil graphite electrode (PGE) as the working electrode, with an Ag/AgCl/3 M KCl (BAS, Model RE-5B, Lafayette, LA, USA) as a reference electrode and a platinum wire as the auxiliary electrode. A Rotring Pencil model (Germany) was used as a holder for the graphite lead (Tombow 0.5 HB, Japan). A metallic wire was solder to the metallic part of pencil to have electrical contact with the lead. The pencil lead was held vertically with 14 mm of the lead protruding outside during each measurement (10 mm of which was immersed into the solution).

### 2.2. Chemicals

The synthetic oligonucleotides were purchased from TIBMOLBIOL (Berlin, Germany). Since the inosine substituted DNA probe sequence-specific to MYC does not contain any guanine base, a label-free (or indicator-free) detection of MYC DNA hybridization could be achieved by measuring the oxidation signal of guanine in the presence of full-match hybridization between inosine substituted DNA probe and its complementary DNA target [23,24,25,26,27]. 

The base sequences of the DNA oligonucleotides used in our study were given as below:

**DNA oligonucleotide (20 base, DNA ODN):**

5′-NH_2_-(CH_2_)_6_-AGG GTG TCT GAA GGA GGG GG-3′

**MYC DNA probe (17 base, I: inosine):**

5′-NH_2_-(CH_2_)_6_-TCA AAT CAI ITT ICT TA-3′

**MYC DNA target (17 base):**

5′-TAA GCA ACC TGA TTT GA-3′

**Noncomplementary DNA oligonucleotide (23 base, NC):**

5′-AAT ACC TGT ATT CCT CGC CTG TC-3′

**Two bases mismatched DNA oligonucleotide (17 base, MM):**

5′-TAA GCA A**GG** TGA TTT GA-3′

The stock solutions of oligonucleotides (500 µg/mL) were prepared with Tris–EDTA buffer solution (10 mM Tris–HCl, 1 mM EDTA, pH: 8.0; TE) and kept frozen. More diluted solutions of amino linked DNA, MYC probe sequences were prepared in 0.5 M acetate buffer solution containing 20 mM NaCl (ABS, pH 4.8). More diluted target/NC/MM solutions or the mixture of target:NC (1:1) and target:MM (1:1) were prepared in 10% diluted SSC (pH 7.0) or 0.02 M Tris–HCl buffer solution containing 20 mM NaCl (TBS, pH 7.0) and 0.05 M phosphate buffer containing 0.5 mM NaCl (PBS, pH 7.4).

IL (1-butyl-3-methylimidazolium hexafluorophosphate) was purchased from Sigma-Aldrich. All other chemicals were of analytical reagent grade and were supplied from Sigma-Aldrich and Merck. All other stock solutions were prepared using ultrapure and deionized water.

### 2.3. Procedure

The immobilization of nucleic acid and its detection cycle was performed using IL modified PGEs. The control experiments were also done using unmodified PGEs. All experiments were carried out at room temperature. 

#### 2.3.1. Preparation of IL Modified PGE

The required amount of IL dissolved in 30 min sonicated organic solvent N,N-dimethylformamide (DMF) solution and then sonicated during an hour at room temperature. PGEs were electrochemically pretreated by applying +1.40 V for 30 s in ABS (pH 4.8). Each pretreated pencil lead was immersed into the eppendorf tubes containing 110 µL of IL during an hour as explained in our previous report [13,27]. Then, the electrodes were then allowed to dry for 30 min at upside position without rinsing.

#### 2.3.2. Microscopic Characterization of Unmodified and IL Modified PGE by Scanning Electron Microscopy (SEM)

The microscopic characterization of bare (unmodified), IL modified PGE was obtained by Quanta 400 FEI, field emission scanning electron microscope (FE-SEM) (Tokyo, Japan) with required acceleration voltage as 5kV with different resolutions 1 to 50 µm.

#### 2.3.3. Electrochemical Monitoring of DNA Hybridization

***Probe DNA Immobilization onto the Electrode Surface***

IL-PGEs were immersed into the vials containing 110 µL of required amount of MYC DNA probe prepared in ABS (pH 4.8) during 1 h. The electrostatic interaction (π-π) occurred between the negatively charged phosphate groups of IL and the positively charged amino group of DNA probe caused by binding of amino linked MYC probe onto the surface of IL-PGE, that was also similarly explained in the literature [27]. Then, each of the electrodes was rinsed with ABS (pH 4.8) for 5 s to remove unbound probe molecules at electrode surface.

***DNA Hybridization at the Surface of MYC DNA Probe Immobilized IL PGE***

Probe immobilized electrodes were immersed into the vials containing required amount of MYC DNA target prepared in an appropriate hybridization buffer medium (10% diluted SSC (pH 7.0), or TBS (pH 7.0), or PBS (pH 7.4) buffer solution) for hybridization process during 1 h. Each of the electrodes was then rinsed with a selected buffer (SSC (pH 7.0), or TBS (pH 7.0), or PBS (pH 7.4) solution) for 5 s. According to the results of experiment related to biosensor performance on hybridization buffer (shown in Figure S1), the appropriate hybridization buffer was selected as SSC (pH 7.0) buffer in our study.

#### 2.3.4. Voltammetric Measurements

Cyclic voltammetry (CV) measurements were performed in 2 mM K_3_[Fe(CN)_6_]/K_4_[Fe(CN)_6_] (1:1) containing 0.1 M KCl by scanning from −0.5 to +1.40 V and the scan rate as 50 mV/s. The raw data was treated using the Savitzky and Golay filter (level 2) of the GPES software.

The differential pulse voltammetry (DPV) measurements were performed in ABS (4.8) for label free electrochemical detection of DNA hybridization between MYC DNA probe and MYC DNA target/NC/MM or the mixture of target:NC (1:1) and target: MM (1:1) by measuring the guanine oxidation signal obtained at +1.00 V by scanning the potential range from (+0.2 V) to (+1.45 V) versus Ag/AgCl/3 M KCl reference electrode at 50 mV pulse amplitude with a scan rate; 50 mV/s. The raw data of voltammograms was also treated using the Savitzky and Golay filter (level 2) of the GPES software, followed by the moving average baseline correction with a peak width of 0.03.

#### 2.3.5. Impedimetric Measurements

The EIS measurements were performed in the presence of 2.5 mM K_3_[Fe(CN)_6_]/K_4_[Fe(CN)_6_] (1:1) mixture prepared in 0.1 M KCl. The impedance was measured in the frequency range from 100 mHz to 100 kHz in a potential of +0.23 V with a sinusoidal signal of 10 mV. The frequency interval divided into 98 logarithmically equidistant measure points. The respective semicircle diameter corresponds to the charge-transfer resistance, R_ct_, the values of which are calculated using the fitting programme AUTOLAB 302 (FRA, version 4.9007, Eco Chemie, The Netherlands).

## 3. Results and Discussion

**Figure 1:**
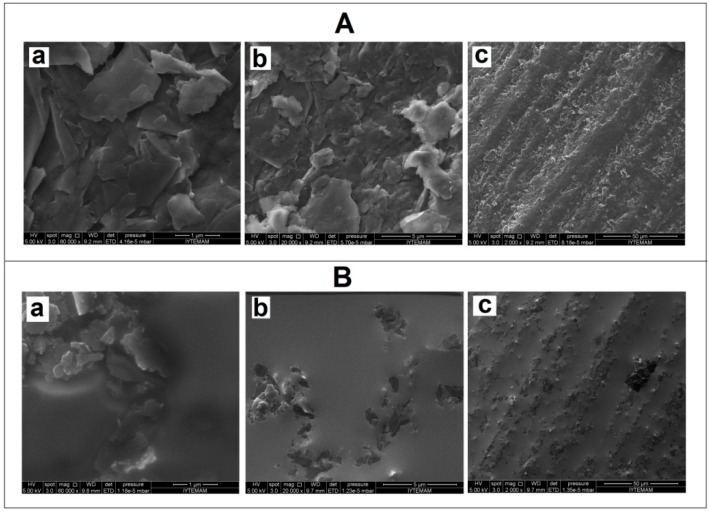
SEM images of unmodified (**A**) and 10% IL modified PGE (**B**) using identical acceleration voltage as 5.0 kV with the resolution 1 to 50 µm (a–c).

The microscopic characterizations of unmodified and 10% IL modified PGEs was performed via SEM technique (Figure 1). The coverage of the layered graphite surface by IL could be clearly seen after the modification of IL (Figure 1A,B). 

**Figure 2:**
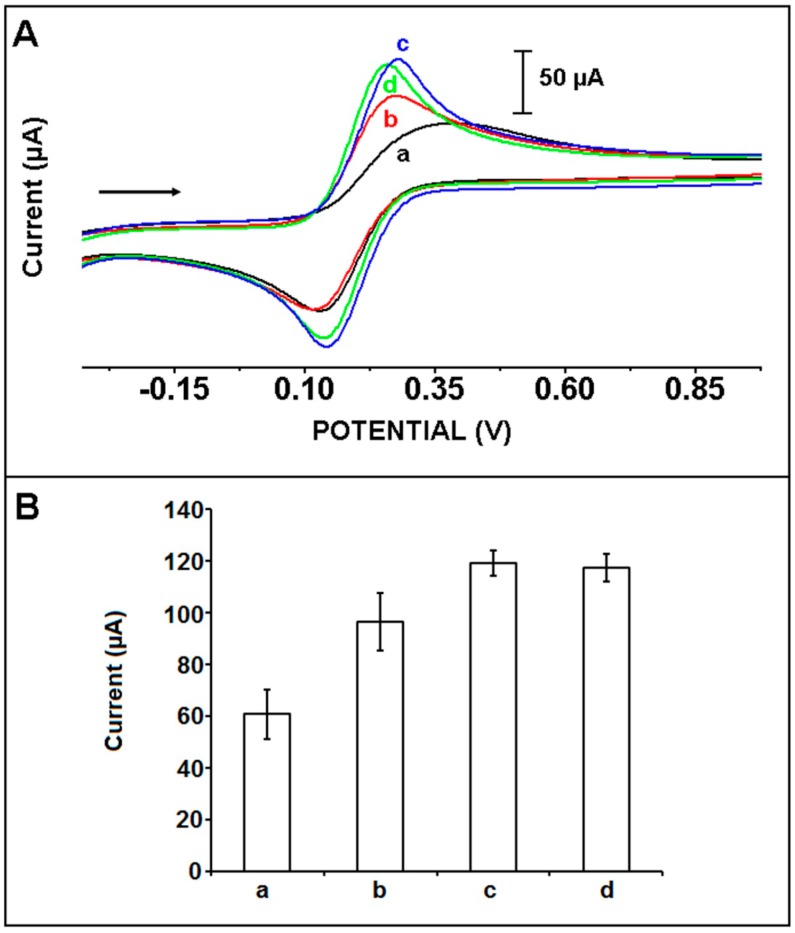
(**A**) The cyclic voltammograms of (a) unmodified, (b) 5%, (c) 10%, (d) 15% IL modified PGE; (**B**) Histograms representing the anodic peak currents (I_a_) obtained by using (a) unmodified, (b) 5%, (c) 10%, (d) 15% IL modified PGE.

Next, the effect of IL concentration onto the modification process was investigated (Figure 2). IL in different ratios was modified onto the surface of PGEs, and the CV measurements were then performed. The average anodic peak current (I_a_) was measured as 60.5 ± 9.7 µA with the relative standard deviation % (RSD %) as 16.1% (n = 11) using unmodified PGEs (Figure 2B(a)). After the modification of 5%, 10% and 15% IL at the surface of PGEs, the average I_a_ was obtained as 96.3 ± 11.2 µA, 118.9 ± 4.8 µA and 117.3 ± 5.2 µA with the RSDs % as 11.7%, 4.1% and 4.4% (n = 6) respectively. After the modification of PGE using IL, the I_a_ increased due to the conductive structure of IL [13,27,28,29]. The highest I_a_ was obtained after the modification of 10% IL at the surface of PGE (Figure 2A,B(c)). Moreover, the anodic charge values (Q_a_), peak to peak separations (∆E_p_) and calculated surface areas (A) of unmodified and IL modified PGEs were calculated (Table S1). The highest Q_a_ and A was obtained after modification of 10% IL onto the surface of PGE which was a result of the increasing of I_a_ value after modification of IL. Moreover, ∆E_p_ decreased after the modification of 10% IL (*i.e.*, 125 mV) compared to the one obtained by unmodified PGE (*i.e.*, 222 mV). This result indicated that the modification of IL at the surface of PGE made the behavior of [Fe(CN)_6_]^3−/4−^ less irreversible [13,27,28,29].

**Figure 3:**
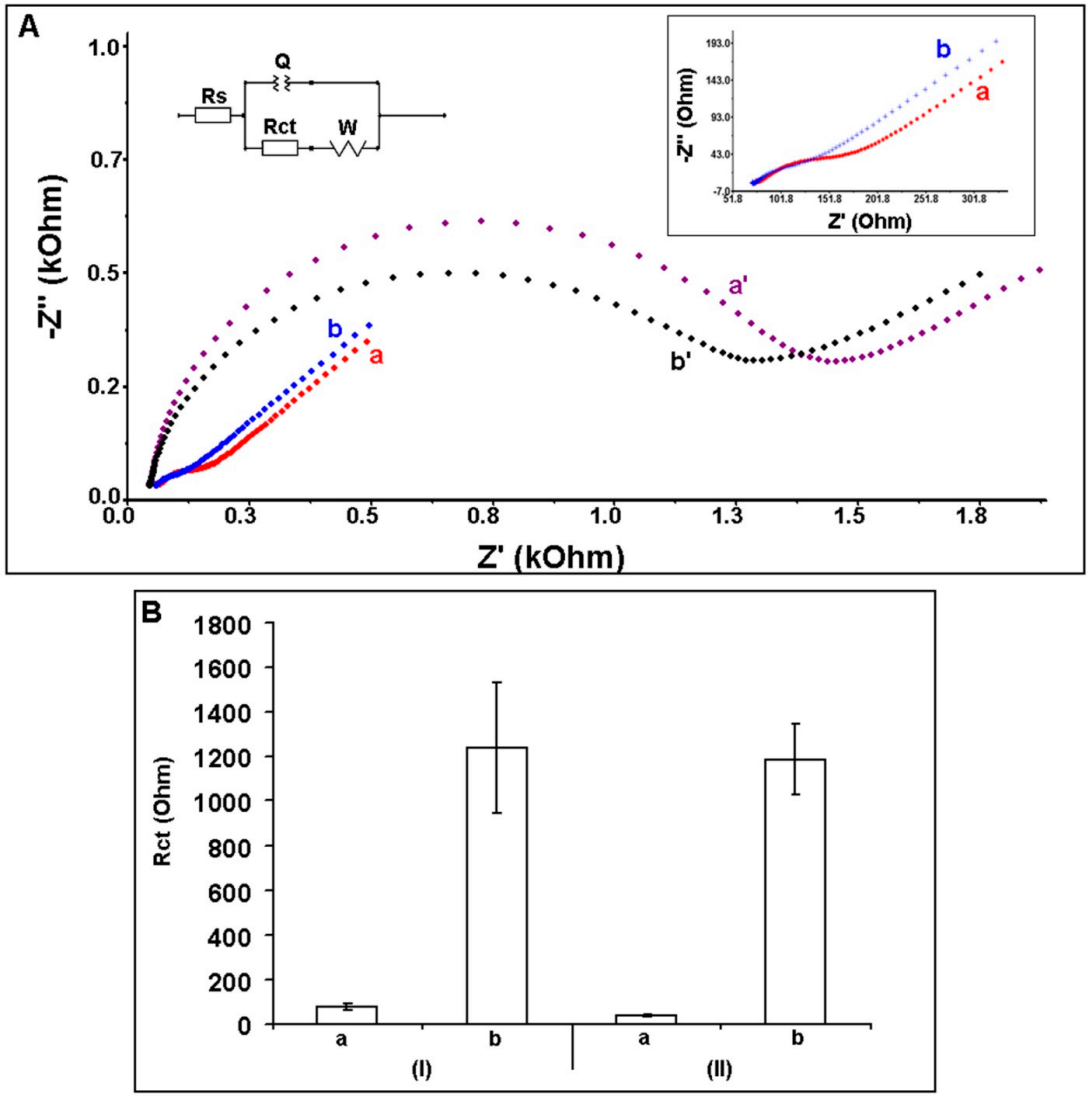
(**A**) Nyquist diagrams representing (a) unmodified PGE, (b) 10% IL modified PGE (a and b also as inset), 100 µg/mL amino linked DNA ODN immobilized (a') PGE, (b') 10% IL modified PGE. Inset was the equivalent circuit model used to fit the impedance data, the parameters of which are listed in the text; R_s_ is the solution resistance. The constant phase element Q is then related to the space charge capacitance at the electrode/electrolyte interface. R_ct_ is related to the charge transfer resistance at the electrode/electrolyte interface. The constant phase element W is the Warburg impedance due to mass transfer to the electrode surface; (**B**) Histograms representing R_ct_ values obtained (a) before and (b) after 100 µg/mL amino linked DNA ODN immobilization of (I) unmodified PGE and (II) 10% IL modified PGE.

The electrochemical characterization of 100 µg/mL DNA oligonucleotide (DNA ODN) immobilized unmodified and 10% IL modified PGEs was performed using EIS technique (Figure 3). The average R_ct_ value was obtained as 80.9 ± 22.1 Ohm (RSD% = 27.3%, n = 9) using unmodified PGEs (Figure 3BI(a)). After the modification of IL onto the surface of PGE, the R_ct_ decreased 52.6% and found as 38.3 ± 7.0 Ohm (RSD% = 18.3%, n = 3, Figure 3BII(a)). This decrease at the R_ct_ may be attributed that the increased conductivity of the PGE surface after the modification of IL [13,27,28,29]. After DNA ODN immobilization at the surface of unmodified and IL modified PGEs, the average R_ct_ values were estimated as 1241.7 ± 294.8 Ohm and 1186.0 ± 157.2 Ohm with the RSDs% as 23.7% and 17.3% (n = 3), respectively. A 14.4 fold increase of R_ct_ was obtained after the immobilization of DNA ODN at unmodified PGEs (Figure 3BI(a,b)), whereas a 30 fold increase of R_ct_ was obtained after the immobilization of DNA ODN at IL-PGEs (Figure 3BII(a,b)). Moreover, more reproducible R_ct_ value could be obtained after immobilization of DNA ODN at IL-PGEs (*i.e.*, 17.3%) compared to the one obtained after immobilization of DNA ODN at unmodified PGEs (*i.e.*, 23.7%). 

The apparent fractional coverage values (Q_IS_^R^) were calculated [42,43] for 100 µg/mL DNA immobilized PGE and IL modified PGE and found to be 0.93 and 0.99, respectively. These values were found in parallel to the impedimetric results indicating the increase binding capacity of DNA at PGE surface in the presence of IL modification. 

**Figure 4:**
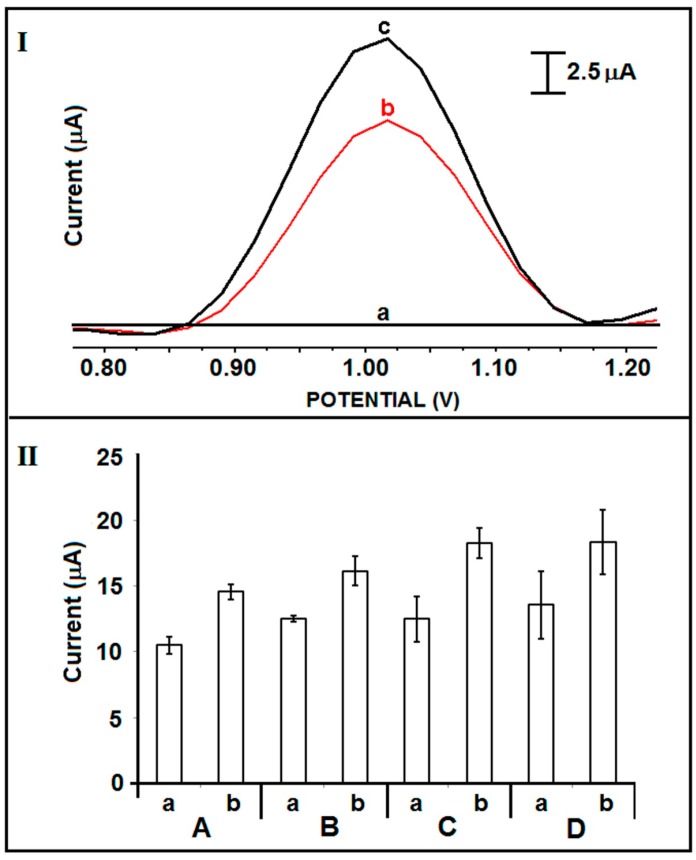
(**I**) Voltammograms representing the (a) control signal coming from IL-PGE, the guanine oxidation signals obtained after immobilization of 15 µg/mL DNA ODN at the surface of (b) unmodified PGE, (c) IL modified PGE; (**II**) Histograms representing the average guanine signals (n = 3) obtained in the presence of (A) 5 (B) 10 (C) 15 (D) 20 µg/mL DNA ODN by using (a) unmodified, (b) IL modified PGE.

The effect of DNA ODN concentration upon the guanine signal was investigated by using unmodified and IL modified PGEs (Figure 4). Amino linked DNA ODN at the concentration level ranging from 5 to 20 µg/mL was immobilized and the increase ratios at the guanine signal obtained by IL-PGEs in contrast to the one obtained by unmodified PGEs were evaluated. The guanine signal obtained by IL-PGEs gradually increased till 15 µg/mL, then it levelled off (Figure 4IIC). Moreover, the guanine signal increased 46% after the immobilization of 15 µg/mL DNA ODN at IL-PGEs in comparison to the one obtained by unmodified PGEs (Figure 4I(b,c),IIC(a,b)). The average guanine signal was measured as 18.2 ± 1.2 µA (RSD% = 6.4%, n = 3) after the immobilization of 15 µg/mL DNA ODN at IL-PGEs (Figure 4IIC(b)). 

**Figure 5:**
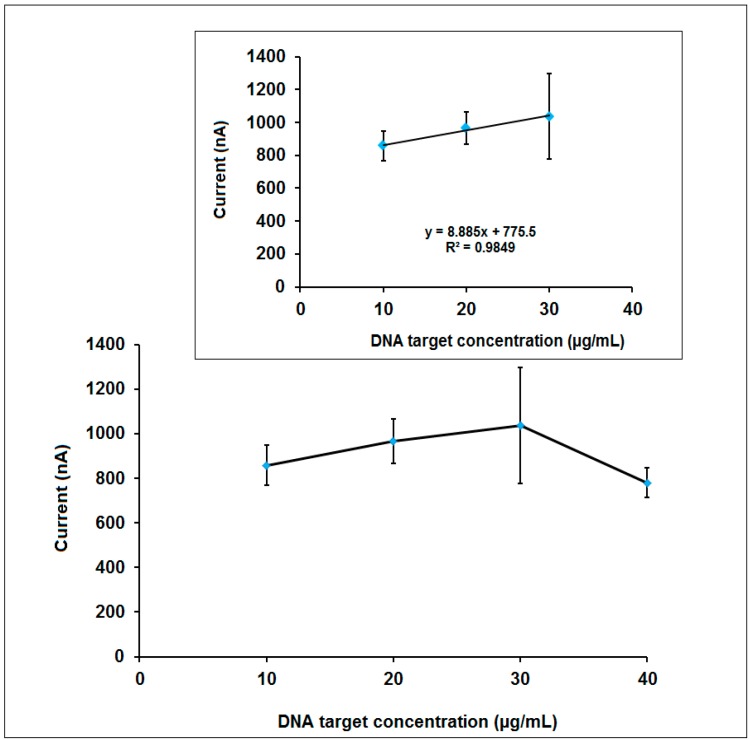
The line graph representing the guanine signal obtained after hybridization of 15 µg/mL MYC DNA probe and its DNA target at the concentration level of 10 to 40 µg/mL. **Inset:** The calibration graph obtained after hybridization of 15 µg/mL MYC DNA probe and its DNA target at the concentration level of 10 to 30 µg/mL.

The effect of MYC DNA target concentration upon the guanine signal obtained after hybridization of 15 µg/mL MYC DNA probe and DNA target at different concentration level from 10 to 40 µg/mL was investigated in the next step (Figure 5). The guanine signal increased till 30 µg/mL MYC DNA target concentration, then it decreased. The detection limit was calculated in the linear concentration range from 10 to 30 µg/mL (Figure 5, inset) using the method described by Miller and Miller [44] and it was found to be 3.72 µg/mL (78.9 pmol in 110 µL sample). Even though the highest guanine signal was obtained after the hybridization of 15 µg/mL MYC DNA probe and 30 µg/mL DNA target, more reproducible guanine signal was obtained in the presence of 20 µg/mL DNA target concentration. Thus, this concentration level was chosen as the optimum one furtherly for selectivity studies. 

**Figure 6:**
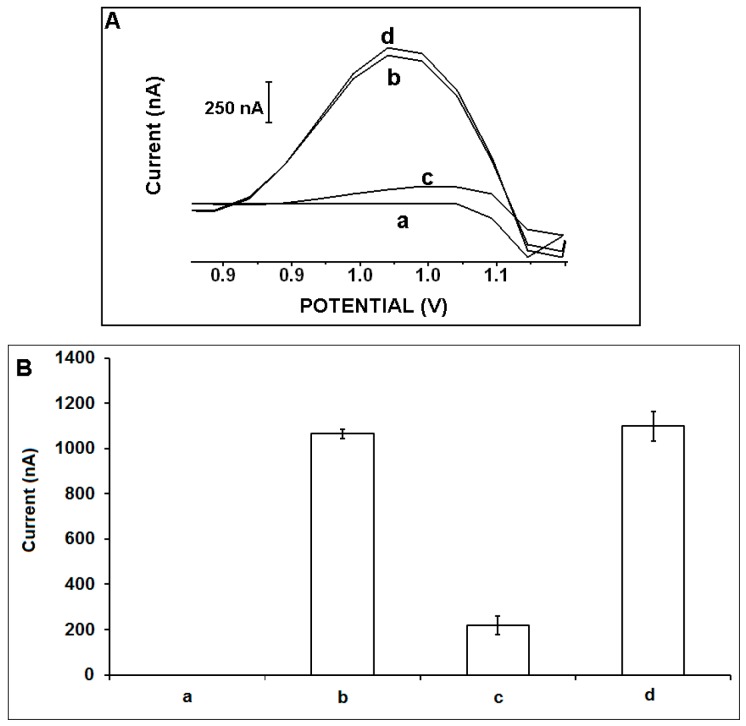
(**A**) Voltammograms, (**B**) histograms representing the guanine signals (n = 4) in the presence of (a) 15 µg/mL MYC DNA probe, after hybridization between 15 µg/mL MYC DNA probe and 20 µg/mL (b) MYC DNA target, (c) NC and (d) the mixture of DNA target:NC (1:1) by using IL-PGEs.

The selectivity of the electrochemical DNA biosensor developed by IL-PGEs was tested in the presence of NC and the mixture of NC: DNA target (1:1) (Figure 6) or MM and the mixture of MM: DNA target (1:1) (Figure S2). After the hybridization of 15 µg/mL MYC DNA probe with 20 µg/mL MYC DNA target, or NC/the mixture of target: NC(1:1), the average guanine signals were measured as 1065.0 ± 19.8 nA, 219.0 ± 39.8 nA and 1098.8 ± 65.9 nA with the RSDs% as 1.9%, 18.2% and 6% (n = 4), respectively. Our DNA biosensor based on the IL-PGEs presented a selective behavior against to NC sequence even if its mixture with DNA target. In the presence of the hybridization between MYC DNA probe and MM, or mixture of MM: DNA target, the average guanine signals were measured as 810.3 ± 132.4 nA and 1613.3 ± 45.7 nA with the RSDs% as 16.3% and 2.8%, respectively. It could be concluded that this voltammetric biosensor based on IL-PGE presented a selective behavior even in the presence of two bases mismatched DNA sequence. 

## 4. Conclusions

In this study, IL modified disposable PGEs were developed and label-free detection of sequence selective DNA hybridization related to *Microcystis* spp. was performed by using IL-PGEs. The microscopic and electrochemical characterizations of IL-PGEs in contrast to unmodified PGEs were successfully done via SEM, EIS and CV techniques, respectively. Under the optimum experimental conditions, the selectivity of our electrochemical DNA biosensor was tested.

The PGEs brought some crucial advantages herein; such as, being disposable, easy to use, time saving (*i.e.*, whole procedure including preparation of IL-PGEs and DNA hybridization was done in 3 h and 45 min), requiring less chemicals and experimental steps comparison to the conventional electrodes used for development of IL modified biosensors [13,21,22,23,24,25,26,27,31,32,33]. Moreover, a low detection limit could be obtained for MYC DNA target by using IL-PGEs. This is the first study in the literature which evaluated the label free voltammetric detection of MYC DNA sequence by using IL modified PGEs.

In conclusion, our biosensing platform could be adapted into the miniaturized systems for electrochemical detection of nucleic acids or, proteins in the future. 

## Supplementary Files

Supplementary File 1
