# Supplementary material for: Development of Ionic Liquid Modified Disposable Graphite Electrodes for Label-Free Electrochemical Detection of DNA Hybridization Related to Microcystis spp
Source: Sensors (Basel). 2015 Sep 9;15(9):22737–49. doi: 10.3390/s150922737 (PMC4610473; doi:10.3390/s150922737)
Supplement: Supplementary File 1 [file sensors-15-22737-s001.pdf]

OPEN ACCESS
sensors
ISSN 1424-8220
www.mdpi.com/journal/sensors

Supplementary Information

Development of Ionic Liquid Modified Disposable Graphite Electrodes for Label-Free Electrochemical Detection of DNA Hybridization Related to *Microcystis* spp. *Sensors* 2015, *15*, 22737-22749

Ceren Sengiz †, Gulsah Congur † and Arzum Erdem *

Faculty of Pharmacy, Analytical Chemistry department, Ege University, Bornova, 35100 Izmir, Turkey; E-Mails: ceren.sengiz@gmail.com (C.S.); gulsah.congur@gmail.com (G.C.)

† These authors contributed equally to this work.

* Author to whom correspondence should be addressed; E-Mail: arzum.erdem@ege.edu.tr or arzume@hotmail.com; Tel.: +90-232-3115131; Fax: +90-232-3885258.

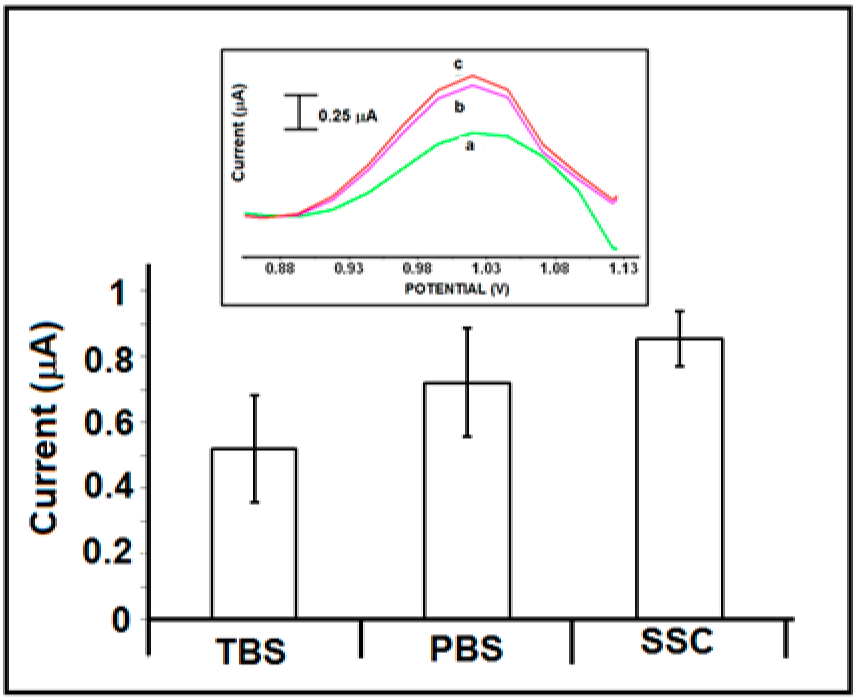

Figure S1. Histograms representing the average guanine signals (n=3) obtained after hybridization of 15 µg/mL MYC DNA probe and 20 µg/mL MYC DNA target in TBS (pH 7.0), PBS (pH 7.4) and SSC (pH 7.0). Inset: The voltammograms representing the guanine signals obtained after hybridization of 15 µg/mL MYC DNA probe and 20 µg/mL MYC DNA target in (a) TBS (pH 7.0), (b) PBS (pH 7.4) and (c) SSC (pH 7.0).

Table S1. The anodic peak currents (I_a), anodic charge values (Q_a), peak to peak separations (ΔE_p) and calculated surface areas (A) of PGE and IL modified PGEs.

	I_a (µA)	Q_a *10^{-4} (Coulomb)	ΔE_p (mV)	A (cm^2)
PGE	62.31	18.1	222	0.188
%5 IL modified PGE	88.30	20.8	144	0.266
**%10 IL modified PGE**	**116.20**	**23.2**	**125**	**0.351**
%15 IL modified PGE	113.60	21.8	117	0.343

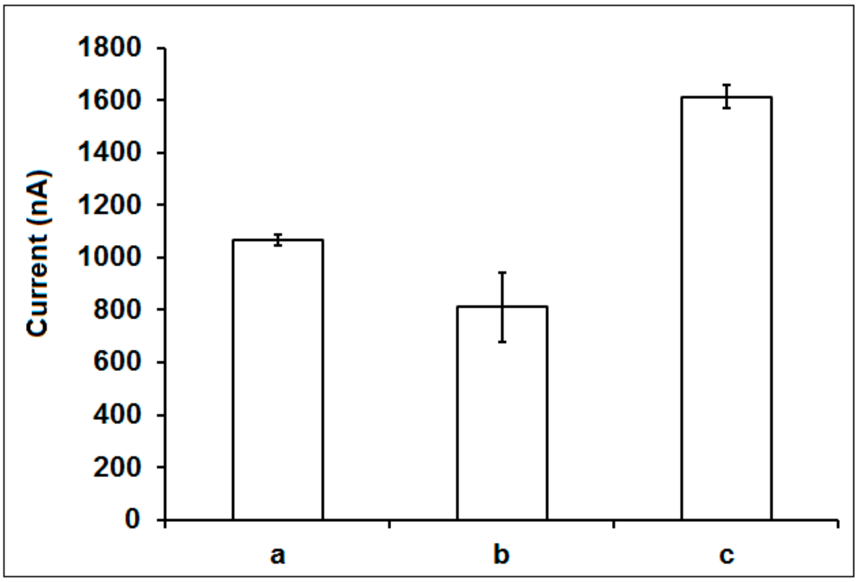

Figure S2. Histograms representing the guanine signals (n=3) after hybridization between 15 µg/mL MYC DNA probe and 20 µg/mL (a) MYC DNA target, (b) MM and (c) the mixture of DNA target:MM (1:1) by using IL-PGEs.

© 2015 by the authors; licensee MDPI, Basel, Switzerland. This article is an open access article distributed under the terms and conditions of the Creative Commons Attribution license (http://creativecommons.org/licenses/by/4.0/).
